# Characterization of the Microstructure of Sr_0.75_Ba_0.25_Nb_2_O_6_ Thin Films by Brillouin Light Scattering

**DOI:** 10.3390/nano14231963

**Published:** 2024-12-06

**Authors:** Alexey Pugachev, Andrey Tumarkin, Sergey Adichtchev, Ludmila Ivleva, Alexey Bogdan

**Affiliations:** 1 Institute of Authomation and Electrometry, Russian Academy of Sciences, 630090 Novosibirsk, Russia; apg@iae.nsk.su (A.P.); adish@iae.nsk.su (S.A.); 2 Department of Physical Electronics and Technology, St. Petersburg State Electrotechnical University “LETI”, 197376 St. Petersburg, Russia; alexey.bogdan98@gmail.com; 3 Prokhorov General Physics Institute, Russian Academy of Sciences, 119991 Moscow, Russia; ivleva@lst.gpi.ru

**Keywords:** strontium-barium niobate films, microstructure, high-temperature annealing, Brillouin light scattering in thin films

## Abstract

Strontium-barium niobate (Sr_x_Ba_(1−x)_Nb_2_O_6_) films can be considered as a promising material for microwave applications due to high dielectric nonlinearity and relatively low losses. Since strontium-barium niobate has a disordered structure that determines its unique electrical properties, the identification of structural features of the Sr_x_Ba_(1−x)_Nb_2_O_6_ films is the key to their successful use. The Sr_x_Ba_(1−x)_Nb_2_O_6_ films were synthesized on a sapphire substrate by magnetron sputtering. The structure of the films was studied by both traditional methods of electron microscopy, X-ray diffraction, and the rarely used for thin films investigation Brillouin light scattering method, which was the focus of our study. We show that Brillouin light scattering is an excellent nondestructive method for studying the structural features of thin ferroelectric strontium-barium niobate films. An analysis of the features of the Brillouin light scattering spectra in thin-film structures and their comparison with the spectra of bulk crystals allowed us to determine with high accuracy the thickness of the films under study and their structural features determined by the resonant scattering of acoustic waves.

## 1. Introduction

Ferroelectrics (FE) exhibit a strong dependence of permittivity on the external electric field with relatively low losses, which makes them promising materials for use in microwave electronics. The dielectric nonlinearity of ferroelectrics allows them to be used as the basis for electrically tunable capacitors, filters, and phase shifters, which are key elements of microwave devices. The most promising option for implementing such elements is a thin-film structure consisting of a dielectric substrate, a ferroelectric film in which large control fields are easily implemented, and electrodes of a certain configuration. An important advantage of such a structure is its ease of implementation and low cost.

Today, the most studied material for use in microwave devices is barium-strontium titanate BaSrTiO_3_ (BST) in the paraelectric state [1,2,3,4,5]. Compared with ferrite materials traditionally used for tunable microwave devices, BST demonstrates a higher response speed [2], and compared with semiconductor materials, a higher level of operating power for a microwave signal [6,7]. Operation at high power levels compared with semiconductor devices is determined by the possibility of creating distributed film structures with a large heat sink area. The material demonstrates microwave electrodynamic properties that are promising for phase-shifting devices and tunable deflectors with operating frequencies up to 40 GHz [8,9].

Nevertheless, the relatively large relaxation times of permittivity and the dielectric loss levels of BST films stimulate the search for alternative ferroelectric solid solutions for microwave applications [10].

Strontium-barium niobate Sr_x_Ba_(1−x)_Nb_2_O_6_ (SBN) seems to be such an alternative to barium-strontium titanate. At x ≥ 0.6, the material belongs to the class of relaxor ferroelectrics, has a disordered structure, and due to this, exhibits a number of unique physical characteristics. Large values of nonlinear optical coefficients and relatively low coercive fields, of the order of 1 kV/cm, characteristic of SBN, make it possible to create elements with regular domain structures on its basis for the creation of efficient frequency converters of laser radiation [11].

In recent years, much attention has been paid to experimental and theoretical studies of the properties of heterostructures based on SBN thin films. These films are of interest for microwave applications for the following reasons:

(a) unlike conventional homogeneous ferroelectrics, the values of dielectric, piezo-, pyroelectric, electrical and nonlinear-optical characteristics in SBN crystals are extremely high, due to which the material has pronounced nonlinearity of dielectric properties and their dependence on external influences [12];
(b) the phase transition and anomalies of the properties of relaxor SBN are significantly blurred over a wide temperature range, which makes strontium-barium niobate a promising thermally stabilized ferroelectric material for microwave applications [13,14];
(c) SBN solid solutions demonstrate a decrease in the values of dielectric relaxation and leakage currents with an increase in the content of strontium atoms, which can have a positive effect on the level of microwave losses of the FE device [15];
(d) the material has high electrical strength, which opens up the possibility of increasing the applied electric field to the tunable element and increasing the dielectric nonlinearity of the microwave device;
(e) the introduction of impurity ions into the real structure of the SBN crystal leads to a significant change in the dynamics of phase transformations, the appearance of new physical properties, and expands the areas of application of such crystals [16,17].

Compared with numerous studies of strontium-barium niobate for applications as electro-optical devices, holographic storage devices and the basis of non-volatile random-access memory, information on the microwave dielectric properties of SBN films is fragmentary. Single works on this topic report on the production of oriented thin SBN films on single-crystal substrates by pulsed laser deposition and gas-discharge RF sputtering. All works use a traditional technological approach, i.e., solve the problem of oriented growth of SBN film on structurally matched substrates (MgO and LaAlO_3_), which are not the optimal choice for microwave applications. Expectedly, such a choice of materials does not lead to the production of structures exhibiting promising microwave characteristics [18,19,20]. Attempts to grow high-structural quality SBN films on substrates in demand in microwave electronics (sapphire, alumina) encounter significant difficulties. The task is complicated by the structural mismatch of the film and substrate materials and the incompletely filled SBN crystal lattice, which has a significant effect on the properties of the material. Since the electrical characteristics of the films directly depend on the quality of the structure, their comprehensive structural analysis becomes necessary.

Various methods are actively used to study ferroelectric crystals and films. X-ray structural analysis is used to study the symmetry and quality of a crystalline film [21,22,23]. Studying the pyroelectric response of a material allows one to determine the values of spontaneous polarization, phase transition temperatures, etc. [24,25]. Dielectric spectroscopy is widely used to study the temperature and frequency dependence of permittivity [20,26]. It should be noted that measurements of the pyroelectric response, hysteresis loops and permittivity at high and ultra-high frequencies require the application of metal contacts. Often depletion layers, Schottky barriers, etc. are formed at the metal-ferroelectric interface. In other words, it is not the film itself that is studied, but the metal–film–metal–substrate structure [27]. In addition, the procedure of applying contacts in most cases introduces irreversible changes in the structure of the ferroelectric film (mutual diffusion of elements, formation of transition layers, depletion of the film in oxygen, etc.). In this regard, the role of contactless, non-destructive, and spatially selective methods for studying ferroelectric materials increases. The most informative are spectroscopic methods, such as Raman and Brillouin light scattering, IR spectroscopy, optical second harmonic generation. Raman and IR spectroscopy analyze the frequency spectra of oscillations of the crystal structure of the material under optical excitation. Using the Raman method (by comparing a film with a crystal of the same chemical composition), it is possible to obtain information about the presence of mechanical stresses, structural defects, etc. However, the Raman spectrum contains only inhomogeneously broadened lines, the temperature behavior of which is difficult to interpret. The parameters describing these lines are weakly sensitive even to the ferroelectric phase transition [17,28]. The method of second harmonic generation is used to study the temperature and polarization dependence of macroscopic spontaneous polarization in the ferroelectric phase [29].

Among spectroscopic methods, Brillouin light scattering (BS) occupies a special place. Studying the frequency shift, width and shape of the Brillouin line, as well as their temperature dependences, allows determining the sound propagation velocity in the crystal, the elastic modulus, and the attenuation coefficient of elastic waves in the microwave range, which directly depend on the structural features of the material—defects, crystallite size and length of grain boundaries. The study of the interaction of electric fields in a crystal with acoustic waves due to the piezoelectric effect or electrostriction makes a great contribution to the study of high-frequency properties of the material [30]. It is believed that Brillouin light scattering is more applicable to bulk crystals and less applicable to films, since the intensity of the scattered signal in the film is significantly less. Moreover, a small film thickness (less than or comparable to the wavelength of light) introduces additional features into the spectral shape of the BS doublet, which significantly complicates the analysis. In this case, when irradiated in the direction perpendicular to the film plane, the traveling wave conditions are not met and the spectral shape of the BS doublet is not a separate narrow peak, as in a bulk crystal, but a set of peaks approximated by the Lorentz function, modulated by the field of the scattered wave [31,32]. This effect, on the other hand, allows us to determine important parameters of the film itself. When the phonon vector propagates along the film plane (the so-called 90A geometry), the doublet shifts to the region of lower frequencies. This fact allows us to distinguish the peaks of the BS doublet from the peaks caused by resonant oscillations of microinhomogeneities [33,34,35], and to answer the question about the presence of inhomogeneities or relaxation processes in the volume of the film [36]. Therefore, when Brillouin light scattering occurs in film structures, the spectrum type in the microwave range can provide information on the structural features of the crystal lattice of the film material, which is critically important for assessing its microwave properties (nonlinearity, dielectric losses, and slow relaxation rate). In addition, the properties of films can differ from the properties of the corresponding crystals due to mechanical stresses at the substrate–film interface, growth defects in the film, features of the film structure [23,37,38,39], etc. Comparison of BS spectra from bulk crystals and films can also reveal features of film structures.

In all the optical methods described above, in contrast to “traditional” ones (X-ray, pyroelectric, and dielectric effects), the possibility of a contactless study of the film in individual local areas with high spatial resolution, limited only by the diameter of the focused exciting laser beam, is realized. In this regard, the aim of this work is to demonstrate the capabilities of the BS method for studying thin films whose structure can change significantly as a result of technological operations. We show that Brillouin light scattering is an excellent non-destructive method for monitoring the properties of ferroelectric films of complex structure. In this work, we study SBN films on sapphire substrates using the Brillouin light scattering method in order to identify the features of their formation as a result of deposition and high-temperature post-growth treatment for their further use in tunable elements of the microwave range.

## 2. Features of Brillouin Light Scattering in Thin Films

The spectral shape of the BS doublet in thin films (we will call films thin if their thickness is comparable to or less than the wavelength of the exciting light) depends significantly on the film thickness. This effect was investigated in [31] on Mn_2_PS_4_ films of submicron thickness, on thin polymer films [32,40], on thin micron silica glass [41], on supported polystyrene, and poly(methyl methacrylate) films [42].

When a film is irradiated with laser radiation in a direction perpendicular to the film plane (it is assumed that the material is homogeneous and there are no losses), the normal modes in the direction perpendicular to the film surface are characterized by the wave vector $K_{⏊}^{m}$, which takes discrete values satisfying the boundary conditions. In particular, for a film deposited on a solid substrate, $K_{⏊}^{m}$ = (2m − 1)/2*d*, where m = 1, 2, 3… and *d* is the film thickness [31].

The allowed acoustic modes are a node at the interface with the substrate and an antinode on the film surface. At normal incidence of laser radiation on the film surface, the quantization of the modes creates a series of equally spaced peaks in the Brillouin spectrum, the distance between which in frequency Δ*f* is related to the speed of sound in the material *u* through the film thickness, i.e., Δ*f* = *u*/2*d*. This comb structure of the acoustic modes in the Brillouin spectra is modulated in intensity by the phase-matching condition with the optical scattered field. In particular, the intensity of each mode $K_{⏊}^{m}$ is expressed by the envelope sin *c*^2^(*x*) centered around q, the wave vector of phonons propagating along the normal to the film plane q = 2π/Λ, where Λ is the phonon wavelength [31]:

(1) $$I_{m}∼\frac{sin^{2}\left[\left(K_{⊥}^{m}-q\right)\frac{d}{2}\right]}{{\left[\left(K_{⊥}^{m}-q\right)\frac{d}{2}\right]}^{2}}$$

where *I*_m_ is the total intensity of scattered light.

In [32], it is shown that *I*_m_ is described by the product of the sum of the Lorentzians *L*_Σ_ and the envelope *S*:

(2) $$I_{m}(\omega )=C+I\cdot L_{\Sigma }S,$$

where

(3) $$L\Sigma =\sum_{m}^{\infty }\frac{\frac{Г}{2}}{\left[{\left(\frac{Г}{2}\right)}^{2}+{\left(\omega -\frac{(2m-1)\Delta f}{2}\right)}^{2}\right]}$$

(4) $$S=\sin c^{2}\left(\frac{\pi }{2\Delta f}\left(\omega -\frac{4\pi nd\Delta f}{\lambda }\right)\right)$$

Here, Г is the width of the Lorentz contour, n is the refractive index of the film, and Δ*f* in the case of backscattering is related to the position of the Brillouin line *f*_b_ by the relation $\Delta f=u/2d=f_{b}/4\pi d\lambda$. In Formulas (2)–(4), Δ*f* (*f*_b_), Г, *d*, and C are adjustable parameters.

**Figure 1:**
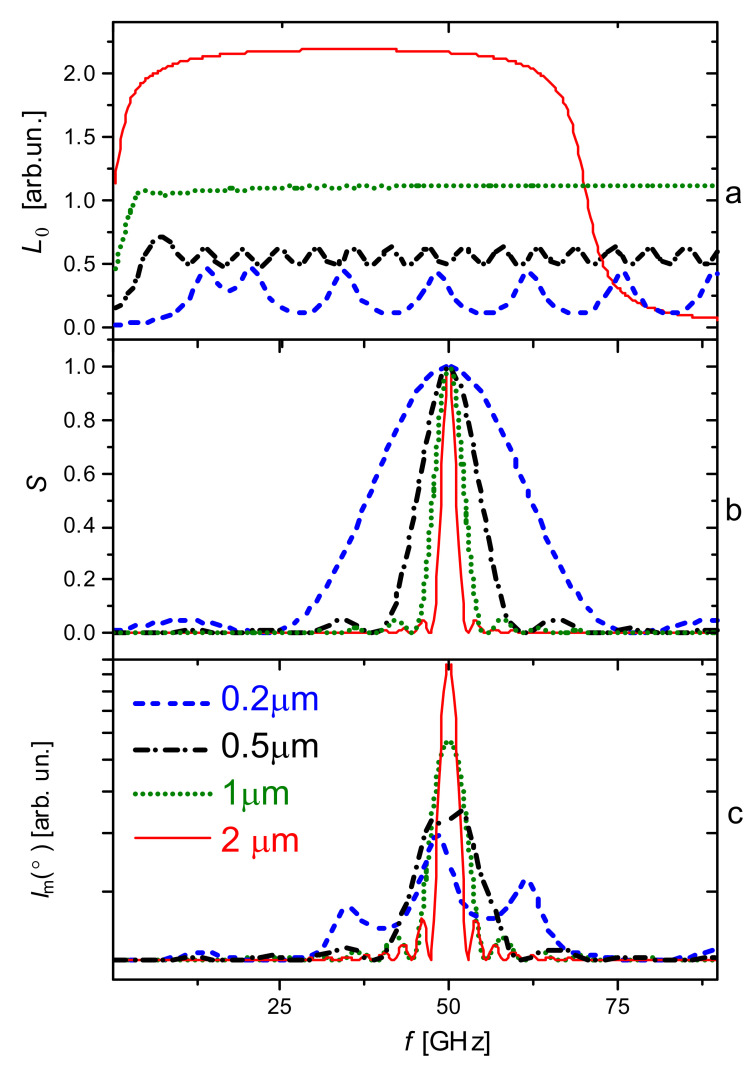
Calculated dependence of the spectral shape of the BS peak depending on the film thickness (0.2–2 µm). (**a**) the calculation of the value of L_Σ_ according to Formula (3), (**b**) the value of *S* according to Formula (4). (**c**) the final result according to Equation (2), presented in a logarithmic scale for better visualization.

Figure 1 shows the calculations using Formulas (2)–(4) of the acoustic response of SBN crystalline films of different thicknesses, where the values of Г ≈ 1 GHz and *f*_b_ ≈ 50 GHz are taken from measurements of bulk SBN 75 and SBN 50 crystals at room temperature [43,44]. It follows from these works that for the above-mentioned samples the values of *f*_b_ at room temperature are close in magnitude. For the calculation, we took the value m = 55, n = 2.2 [45], λ = 532 nm. In the future, the experimental spectra will be compared with these calculations.

Let us emphasize the features of BS in thin-film structures that are important for obtaining information about the structure of the film under study. First, if the film thickness is comparable to the wavelength, then according to Formula (1), the width and structure of the longitudinal mode peak in the BS spectrum will depend significantly on the film thickness. Second, when films are excited by laser radiation in the gigahertz range, not only BS doublets can appear. If the film has a complex spatial structure (in particular, consists of submicron particles), the BS spectra show lines of resonant scattering of acoustic waves on these particles. The spectral shape and frequency of these lines are determined by the shape, size of the particles, and the spectral scattering geometry [46]. Thus, the thickness of the film under study and its structural features can be estimated from an analysis of the spectrum shape, frequency positions of the peaks and their broadenings in various BS geometries.

## 3. Materials and Methods

### 3.1. Synthesis of SBN Thin Films

The SBN thin films were synthesized by RF magnetron sputtering of a Sr_0.75_Ba_0.25_Nb_2_O_6_ (SBN 75) target. The SBN 75 powder was prepared by solid-phase synthesis (temperature 1200 °C, time 12 h) from high purity grade SrCO_3_, BaCO_3_, and Nb_2_O_5_ oxides taken in the stoichiometric ratio and then pressed into tablets 76 mm in diameter and 5 mm thick. Monocrystalline (sapphire) aluminum oxide plates with dimensions of 10 × 10 mm and a thickness of 0.5 mm (produced by MONOCRYSTAL, Stavropol, Russia) were used as substrates for the growth of SBN films. The r-cut of sapphire, which is closest in lattice parameters to the tetragonal SBN 75 crystal, was chosen as a single-crystal substrate.

The film deposition conditions were as follows: the vacuum chamber was pre-evacuated to a pressure of 10^−5^ Pa, pure oxygen was used as the working gas; the target-substrate distance was 25 mm; the discharge current during sputtering was 180 mA, the discharge power was 100 W. The deposition of the SBN films was started at 10 Pa, which was reduced to 2 Pa for the first 30 min of growth. SBN films were deposited for 4 h—the thickness of the films was 700 nm. The temperature of the substrate during the deposition was 950 °C; the substrate was heated by a resistive heater. Such a high film deposition temperature was chosen for the reasons described in [47]. After the deposition process was completed, the films were cooled in an oxygen environment at atmospheric pressure at a rate of 3 °C/min. After cooling to room temperature, the samples were annealed in air at atmospheric pressure for 30 and 60 min at an annealing temperature of 1150 °C. The heating rate of the samples was 10 °C/min and the cooling rate was 5 °C/min.

The film grown on sapphire and not subjected to annealing is hereinafter referred to as sample N1. Films annealed for 30 min and 60 min are referred to as samples N2 and N3, respectively. To analyze the effect of film thickness on the shape of the BS spectrum, a significantly thicker SBN film (sample N4) was also studied.

### 3.2. Structure Investigation Techniques

To study the real structure and composition of the films formed, we used the methods of scanning electron microscopy (SEM), X-ray diffraction analysis (XRD), and Brillouin light scattering. The morphology of samples was studied using SEM. The sample surface was investigated using a TM 3000 Hitachi scanning electron microscope (Tokyo, Japan). The microscope was used under low vacuum conditions; the images were obtained in the back-scattered electrons mode. The diffraction patterns were recorded on a DRON-6 diffractometer (Burevestnik, St. Petersburg, Russia). The recording regimes were as follows: CuK_α_ radiation, K_β_ filter, U = 30 kV, I = 20 mA, Bragg–Brentano geometry, scanning range 2θ = 20°–60°, continuous mode 2°/min. The spectra were identified using the PDF-2 database.

### 3.3. Brillouin Light Scattering Investigation Technique

**Figure 2:**
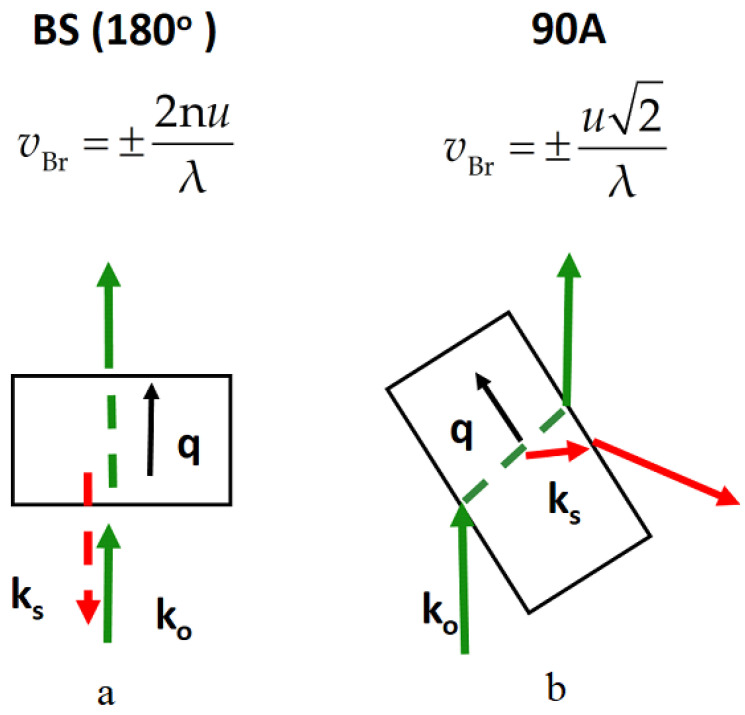
Spectral geometries of the Brillouin light scattering ((**a**) 180° and (**b**) 90 A). Above each diagram are given the formulas for the dependence of the frequency of the BS peak position on the sound velocity and the refractive index. The green and red arrows are the vectors of the exciting and scattered waves.

Elastic properties of the samples were investigated using a Brillouin light scattering. A solid-state laser (average power of 80 mW, wavelength of λ = 532 nm) was used for excitation of light scattering. The spectra were recorded on a six-pass Fabry–Perot interferometer (JRS). The spectral resolution of our experiments was found to be 1 GHz and agrees well with a measurement of emission spectrum of a neon-discharge lamp. The line shift in the Brillouin light scattering was measured in two scattering geometries shown in Figure 2. In the figure, k_o_ and k_s_ are the vectors of the exciting and scattered waves. When the BS is excited, the condition k_o_ − k_s_ = q is satisfied.

1. Backscattering (the angle between the vectors k_o_ and k_s_ is 180 degrees). In this case, the BS line shift $f_{b}=\frac{2nu}{\lambda }sin(\theta /2)$, where θ is the angle between incident and scattered vectors. When θ=180°, $f_{b}=\frac{2nu}{\lambda }$. To measure the signal directly from the film, experiments were carried out in confocal mode. For this purpose, the light beam was focused onto the sample using a micro lens with a numerical aperture of NA = 0.5. After the collimator, the scattered light is focused onto the entrance diaphragm of the spectrometer. Independent measurements showed that the spatial resolution in the experiment did not exceed 1 µm.
2. The geometry 90A, described in more detail in [48], is shown in Figure 2b. A characteristic feature of this geometry is that the vector q propagates along the plane of the film. When the normal and lateral sound velocities are equal, the position of the Brillouin line is determined by a relation independent of the refractive index n: $f_{b90A}=\frac{2u}{\lambda }sin\left(\theta /2\right).$ In this case, the position of the Brillouin peak in strontium-barium niobate (n=2.2) should shift “to the left” relative to the backward scattering in the ratio $\frac{f_{b}}{f_{b90A}}\approx 3$. Deviations from this ratio (changes in the position and width of the peak) can be caused by the anisotropy of the sound velocity in the film.

## 4. Results and Discussion

### 4.1. Structural Analysis of SBN Thin Films

**Figure 3:**
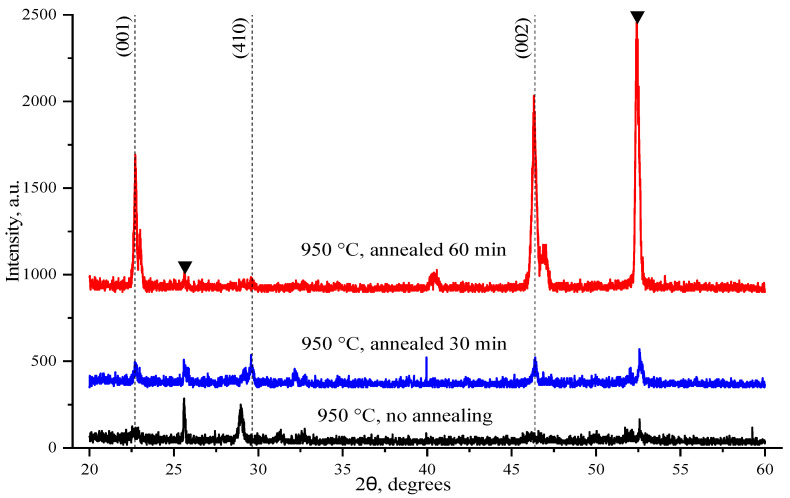
Comparative diffraction patterns of SBN 75 thin films without annealing and after annealing at 1150 °С for 30 and 60 min.

The diffraction patterns of the SBN films before and after annealing are shown in Figure 3. Vertical dotted lines indicate the angular positions of the main reflexes of the solid solution Sr_0.75_Ba_0.25_Nb_2_O_6_ (PDF 73-487), the reflexes of the substrate Al_2_O_3_ are indicated by black triangles. The diffraction pattern of the film before annealing shows weak-intensity reflections of the tetragonal SBN phase and a difficult-to-identify peak at 28.96°, which may belong to either a SBN solid solution (for example, a monoclinic phase [49]) or secondary SrNb_2_O_6_ phase (PDF 45-227). Significant phase transformations are observed in the SBN film on sapphire subjected to annealing. In the case of the film annealed for 30 min, the experimental diffraction pattern has a shape typical for tetragonal films of SBN solid solutions [11] and is characterized by the presence of reflections (001) at 2θ = 22.69° and (002) at 2θ = 46.32°. An increase in the annealing time to 60 min causes a pronounced increase in the intensity of reflections. Thus, the formation of SBN films of high structural quality on sapphire occurs as a result of high-temperature annealing at a temperature of 1150 °C and a duration of 60 min. The crystallite sizes in the samples investigated were estimated from the coherent X-ray scattering region in the film using the Scherrer equation for a tetragonal crystal lattice. For the annealed films, the crystallite size was estimated as 200–250 nm. The uncertainty in estimating the crystallite sizes was about 30 nm. Thus, significant structural differences are observed in the films before and after annealing, which can affect the BS in them.

### 4.2. Brillouin Light Scattering in SBN Thin Films

**Figure 4:**
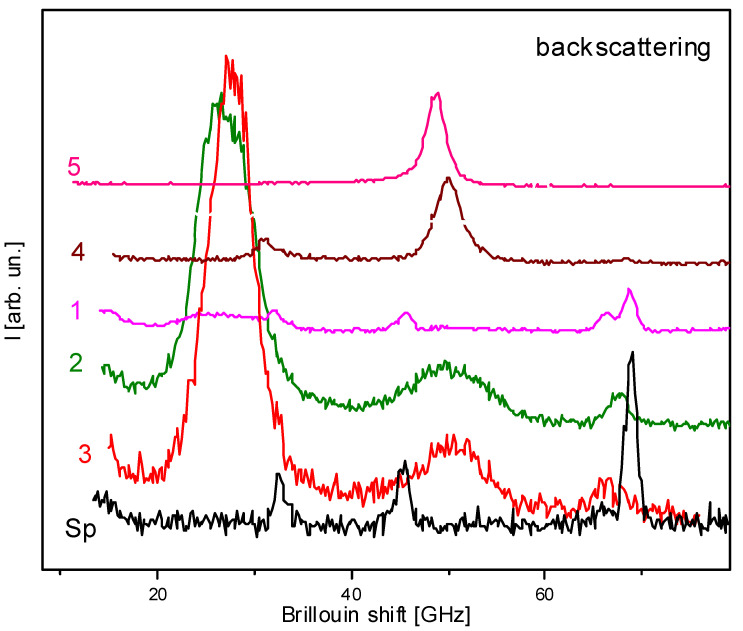
Stokes components of the BS in the backscattering geometry for films (samples N1–N4), designated on the graph by the numbers “1–4” and for the bulk crystal SBN 75 in the Z(XX)$\overset{-}{Z}$ geometry—“5”, Sp—Brillouin spectrum from sapphire substrate.

Typical BS spectra in the backscattering mode in films N1–N4 (Stokes component) are shown in Figure 4. All graphs are normalized and shifted vertically for visualization.

For comparison, the figure shows the spectrum of the bulk SBN 75 crystal in the spectral geometry Z(XX)$\overset{-}{Z}$ designated in Figure 4 by the number “5” (here the first and last letters are the incident and scattered wave vectors of light; in brackets are the corresponding polarizations of the scattered and incident waves; $\overset{-}{Z}$ denotes the direction opposite to Z). According to the selection rules [50], one longitudinal mode (LA) is allowed for this scattering geometry. The appearance of the transverse mode (TA) may be due to a violation of the selection rules due to a discrepancy between the real spectral geometry and the previously declared one (due to inaccurate orientation of the sample, scattering or multiple reflections of light, etc.). The peak in the vicinity of 68 GHz is due to the BS on the sapphire substrate (curve “Sp”). The peak next to it (in the vicinity of 65 GHz) may also be due to either light scattering on the sapphire substrate or the above-mentioned violation of the selection rules. This peak is absent in the bulk crystal (curve 5), and is practically indistinguishable from the background in a relatively thick film (curve 4).

Let us analyze the BS spectra of SBN films with different microstructures. When the film is oriented in the [001] direction, the Brillouin shift is due to the elastic modulus $C_{33}$= ρ$u_{3}^{2}$, where ρ is the density of the material, $u_{3}$ is the velocity of longitudinal sound waves in the direction of the polarization axis (in the gigahertz range). The coincidence of the position of the LA mode peak in the studied films with the corresponding peak in the SBN 75 crystal in the scattering geometry Z(XX)$\overset{-}{Z}$ indicates that the Z axis in these films is directed along the normal to the film plane. The following features in the BS spectra should be noted:

- In the geometry where the wave vector q∥Z in a crystal with 4mm symmetry, according to the selection rules, only the LA mode should be observed, whereas at q⊥Z both LA and TA modes appear. The appearance of the TA mode (*f* ≈ 30 GHz) in sample N4 can be due to a violation of the selection rules, when, due to sharp focusing of the radiation, the exciting light partially propagates at an angle to the Z axis, and the effects of light scattering on defects or multiple light reflections are strong.
- In the spectrum of the unannealed film (N1), the peak of the longitudinal mode is not observed. This confirms the conclusion from the XRD data that in the as-deposited film the SBN solid solution is practically not formed (see Figure 3).
- The width of the longitudinal mode peak in thin films is significantly wider than the corresponding peak in crystals (N2, N3, N4).
- In thin annealed films, an anomalously large peak is recorded in the vicinity of 25 GHz (N2 and N3). To understand the nature of this peak, additional studies were carried out in this work.

### 4.3. Longitudinal Mode in Thin Films in Backscattering Geometry

**Figure 5:**
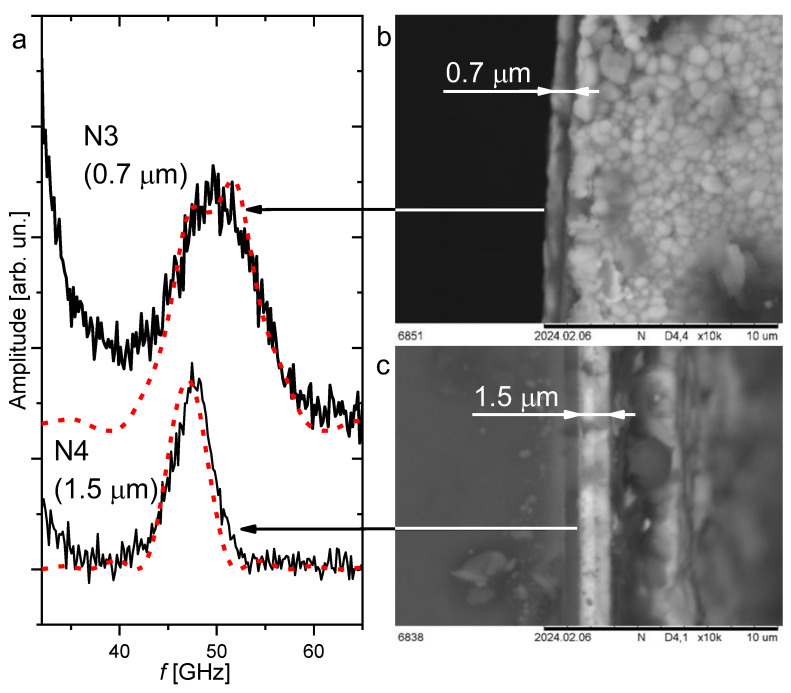
(**a**) Fittings of the spectral shape of the LA mode in samples N3 and N4 according to Formula (1); the numbers in brackets indicate the film thickness obtained from the fittings; (**b**,**c**) are the images of the end of films N3 and N4 obtained on a scanning electron microscope.

As noted earlier, with decreasing film thickness (when it becomes comparable with the wavelength), the width and structure of the longitudinal mode peak change significantly (see Figure 1). Thus, the spectral shape *I*(ω) of the LA mode can be described using the film thickness *d* as a free parameter. Figure 5a shows the fits (red dotted line) of the LA mode spectral shape in samples N3 and N4 using Formula (1) in order to determine the thicknesses of the films under study (the spectral shapes in samples N2 and N3 are identical). For films N3 and N4, the thickness values *d* ≈ 0.7 and 1.5 µm, respectively, were obtained by fitting the spectral shape of the LA mode. We compared the thicknesses obtained from the fitting with the electron microscopy data. Figure 5b,c show the images of the ends of the samples under study, obtained with a scanning electron microscope, from which the film thickness is determined. A comparison of the results allows us to conclude that the data obtained by fitting the spectral shape of the LA mode line using the model described in [31,32] correspond to the thickness obtained from measurements on a scanning electron microscope. When comparing the film thickness determined from Formula (1) and from measurements on a scanning electron microscope, it is important to know the measurement uncertainties and their nature. The greatest uncertainty in measuring the film thickness on an electron scanning microscope is determined by the unevenness of its boundaries. This error can be estimated as the difference between the average thickness and its maximum and minimum values. This value for the films shown in Figure 5b,c is 100 nm. The spectral curve was fitted using Formula (1) in the Origin program. In this case, the fitting error, carried out using the least squares method, was estimated as 80 nm. Thus, it can be concluded that BS can be used as an effective non-destructive method for determining the thickness of thin-film coatings.

### 4.4. The Nature of the “New” Peak in the Vicinity of 25 GHz

**Figure 6:**
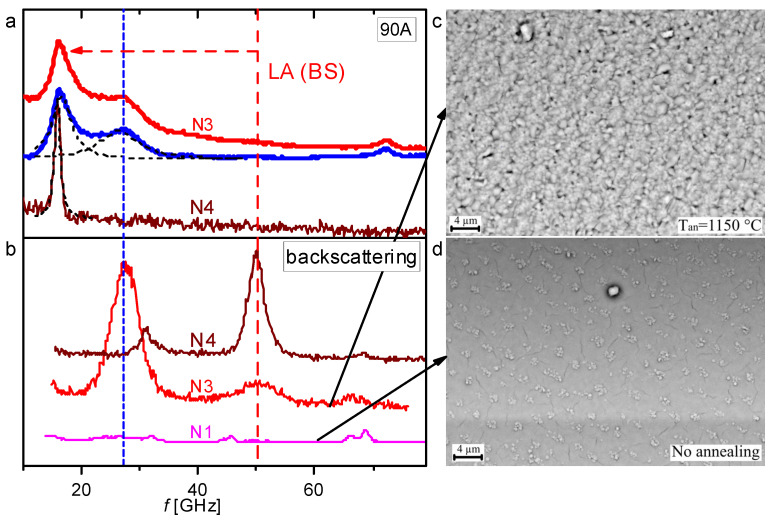
(**a**) The experimental scattering curves from samples N3 and N4 in the 90A geometry. The blue curve is the experimental dependence for sample N3, from which the broadband background was subtracted and the peaks were fitted with two Lorentzians (black dotted lines); the red vertical dotted line indicates the position of the LA mode measured in the backscattering geometry, and the red dotted arrow indicates the position to which this peak has shifted when measured in the 90A geometry; the blue vertical dotted line indicates the position of the “new” peak in the backscattering geometry; (**b**) The experimental BS dependences in the backscattering geometry in samples N1, N3 and N4; (**c**,**d**) show micrographs of the studied films.

It follows from Figure 4 that an abnormally large and wide peak appears in the BS spectrum for SBN 75 films annealed in air at 1150 °C in the vicinity of 25 GHz, in contrast to the spectrum for the film that was not annealed. To clarify the nature of this peak, the BS spectra of the films under study were measured in the 90A geometry. It was shown above that the position of the peaks in the Brillouin scattering in this geometry shifts “to the left” compared to the position of the peak measured in the backscattering geometry. If the peak under study does not change its position, it cannot be interpreted as a BS doublet. Figure 6a shows the BS spectra of films N3 and N4 in the 90A geometry. For comparison, Figure 6b shows the BS spectra of the same films and film N1 in the 180° geometry. The red vertical dotted line indicates the position of the LA mode measured in the backscattering geometry, and the red dotted arrow indicates the position to which this peak has shifted when measured in the 90A geometry. The blue vertical dotted line indicates the position of the “new” peak (25 GHz) in the backscattering geometry.

Thus, in the 90A geometry, the LA mode peak is shifted to a position in the vicinity of 17 GHz, close to the calculated value. The fitting of the spectrum by Lorentzians in the 90A geometry (blue curve in Figure 5a) shows that the width of the LA mode peak in the 90A geometry is about 6 GHz (the broadband background was subtracted from the experimental curve to fit the peak). The broadening of the LA mode peak in the 90A scattering scheme relative to the width of a similar peak in the backscattering mode can be explained by the presence of scattering centers in the bulk of the annealed film, in contrast to the as-grown film.

It also follows from Figure 6 that the position of the peak in the vicinity of 25 GHz in the two studied spectral geometries does not change. The peak width also does not undergo significant changes (in different geometries it ranges from 8 to 10 GHz). This indicates that the nature of the new peak at 25 GHz cannot be interpreted as a BS doublet. It can be assumed that the occurrence of this peak in the BS spectrum of SBN films subjected to high-temperature annealing is associated with the acoustic resonant excitation of submicron particles in the scattering volume. Such an approach has been implemented in a number of works devoted to the study of BS spectra in the GHz range on submicron particles [33,34,35,42]. Thus, it was shown in [33,34] that different types of vibrations of isolated gold particles of 5 nm in size lead to the appearance of relatively broad peaks in the vicinity of 100 and 200 GHz. Moreover, the peak in the vicinity of 200 GHz consists of at least two contributions due to different types of vibrations of nanoparticles. In [35], resonant scattering from harmonics of quadrupole and spherical vibration modes of AgAu nanoparticles in the spectral range from 150 to 450 GHz was detected using the method of high-resolution Raman scattering at low frequencies. In our case, the ensemble of particles was not specially created, but formed spontaneously as a result of high-temperature annealing. We evaluated the structural transformation of the SBN films on sapphire and the change in the crystallite sizes during annealing using electron microscopy and X-ray diffraction analysis [47]. Analysis of the SEM data allows us to conclude that the film structure changes significantly after high-temperature treatment. Moreover, the annealing temperature affects the microstructure: in films annealed at 1100 °C, the crystallite size is about 100–150 nm, while in films annealed at 1150–1200 °C, the grain sizes increase to 250 nm. The XRD analysis data (evaluation of the coherent scattering region of X-rays in a sample) confirm an increase in the crystallite sizes from 100 to 250 nm with an increase in the annealing temperature [47]. Thus, the position that in our case the annealed film may consist of an ensemble of submicron crystallites is confirmed by the data of its structural analysis. Now, let us estimate the sizes of particles that may be the cause of resonant light scattering based on the BS data. In our case, the size of these particles ξ can be estimated based on ξ ≈ *u*/*f*_b_. From the experiment *f*_b_ ≈ 25 GHz, and according to *u* = *f*_b_λ/2n, for SBN film *u* ≈ 6000 m/s. From here, we can estimate the value ξ ≈ 250 nm. This assessment can be confirmed by the SEM data presented in Figure 6. Electron microscopy studies of SBN 75 films showed that films on sapphire substrate without annealing are relatively homogeneous. Annealing of films results in the appearance of a microstructure in the film with approximately the same granule sizes. It can be assumed that conditions for resonant oscillations of granules of this size are created in the annealed films, which explains the occurrence of an anomalous peak at a frequency of 25 GHz. It should be noted that oscillations of particles of significantly larger and smaller sizes can lead to the appearance of resonant peaks in relatively low-frequency (below 10 GHz) and high-frequency (more than 100 GHz) ranges, which is the subject of further research.

## 5. Conclusions

The SBN films grown on sapphire by magnetron sputtering were studied by the Brillouin light scattering, X-ray diffractometry, and electron microscopy methods. The results of the studies demonstrate that high-temperature post-growth annealing significantly changes the film structure. These changes are identified for the studied films with the BS method for the first time. When studying the SBN films with the BS method, it was shown that the thickness of the film under study can be estimated based on the width and structure of the peak of the longitudinal BS mode. A comparison of the results allows us to conclude that the film thickness obtained by fitting the spectral shape of the LA mode line correspond to the thickness measured by a scanning electron microscopy method. Analysis of the XRD and SEM data and BS spectra for annealed and non-annealed films shows that the films subjected to annealing contain a submicron structure with grain sizes of about 250 nm. Resonant acoustic excitation of submicron particles leads to the appearance of an anomalous peak in the BS spectrum, which allows identifying the structural features of the studied films.

## Data Availability

The data are contained within the article.
